# The LORF5 Gene Is Non-essential for Replication but Important for Duck Plague Virus Cell-to-Cell Spread Efficiently in Host Cells

**DOI:** 10.3389/fmicb.2021.744408

**Published:** 2021-12-02

**Authors:** Bingjie Shen, Yunjiao Li, Anchun Cheng, Mingshu Wang, Ying Wu, Qiao Yang, Renyong Jia, Bin Tian, Xumin Ou, Sai Mao, Di Sun, Shaqiu Zhang, Dekang Zhu, Shun Chen, Mafeng Liu, Xin-Xin Zhao, Juan Huang, Qun Gao, Yunya Liu, Yanling Yu, Ling Zhang, Leichang Pan

**Affiliations:** ^1^Institute of Preventive Veterinary Medicine, Sichuan Agricultural University, Chengdu, China; ^2^Key Laboratory of Animal Disease and Human Health of Sichuan Province, Sichuan Agricultural University, Chengdu, China; ^3^Avian Disease Research Center, College of Veterinary Medicine, Sichuan Agricultural University, Chengdu, China

**Keywords:** duck plague virus, LORF5 gene, virus replication, non-essential, cell-to-cell spread

## Abstract

Duck plague virus (DPV) can cause high morbidity and mortality in many waterfowl species within the order Anseriformes. The DPV genome contains 78 open reading frames (ORFs), among which the LORF2, LORF3, LORF4, LORF5, and SORF3 genes are unique genes of avian herpesvirus. In this study, to investigate the role of this unique LORF5 gene in DPV proliferation, we generated a recombinant virus that lacks the LORF5 gene by a two-step red recombination system, which cloned the DPV Chinese virulent strain (DPV CHv) genome into a bacterial artificial chromosome (DPV CHv-BAC); the proliferation law of LORF5-deleted mutant virus on DEF cells and the effect of LORF5 gene on the life cycle stages of DPV compared with the parent strain were tested. Our data revealed that the LORF5 gene contributes to the cell-to-cell transmission of DPV but is not relevant to virus invasion, replication, assembly, and release formation. Taken together, this study sheds light on the role of the avian herpesvirus-specific gene LORF5 in the DPV proliferation life cycle. These findings lay the foundation for in-depth functional studies of the LORF5 gene in DPV or other avian herpesviruses.

## Introduction

Duck plague (DP), also known as duck virus enteritis (DVE), is an acute, febrile, septic, and contagious disease in birds within Anseriformes (such as ducks, geese, and swans). The pathological features of DP include hemorrhagic lesions in the blood vessels, gastrointestinal mucosa, and lymphoid tissues (Dhama et al., 2017). The incidence and mortality of infected ducklings or unprotected adult ducks reach up to 100%, resulting in substantial economic losses for the global waterfowl industry (Yuan et al., 2007; Qi et al., 2008; Xuefeng et al., 2008). Vaccines are considered the most effective means for preventing DP. Live attenuated vaccines of DP virus (DPV) have been used to treat this disease (Qi et al., 2009; Shen et al., 2010; Yang et al., 2010; Huang et al., 2014), and a variety of new effective vaccines have also been developed in recent years (Lian et al., 2011; Yu et al., 2012; Sun et al., 2013), which efficiently control DP but have not completely eradicated it.

Analysis of DPV, the pathogen of DP, may provide insights for the prevention and control of DP. DPV belongs to the α-herpesvirus subfamily and is a double-stranded linear DNA virus (Guo et al., 2009; Xiang et al., 2012; Wu et al., 2018). The genome sequence of the DPV Chinese virulent strain (DPV CHv) was obtained through genome sequencing; the genome has a structure that is typical of α-herpesviruses, except for a lack of terminal repeats (TRS) at the 5′ end (Wu et al., 2012a, b; You et al., 2017). DPV is a cell-free virus with two ways to spread to uninfected cells after replication in infected cells. Cell-free spread occurs when virions are released from an infected cell into its surrounding environment prior to entering a new cell. In addition, all α-herpesviruses, including DPV, also have a “cell-to-cell” spreading mechanism by which virions pass directly through cell junctions to achieve infection of adjacent cells (Farnsworth and Johnson, 2006), enabling escape from neutralizing antibodies (Johnson and Huber, 2002; Mateo et al., 2015). Previous studies have shown that viral genes that are involved in cell-to-cell spread and immune evasion but are non-essential for replication are preferred targets for α-herpesvirus gene-deletion vaccines, such as gE and gI (Vannie et al., 2007; Ndjamen et al., 2014; Weiss et al., 2015; Zhang et al., 2015; Dong et al., 2017). Therefore, clarifying the gene characteristics and functions of DPV would be informative in the prevention of DP.

Currently, the properties of some DPV genes have been characterized, including UL41 (He et al., 2018), US10 (Zhang et al., 2017), UL54 (Liu et al., 2015, 2016, 2017; Gao et al., 2017), UL24 (Gao et al., 2017), UL13 (Hu et al., 2017), US2 (Gao et al., 2015), UL49.5 (Lin et al., 2013, 2014), UL44 (Sun et al., 2014), UL27 (Wang et al., 2011), UL29 (Cheng et al., 2012), and UL16 (He et al., 2012). However, some genes have not yet been studied, such as LORF5, which is located in the UL region of the DPV CHv genome. LORF5 has 723 bp and encodes 241 amino acids. Because the LORF2, LORF3, LORF4, LORF5, and SORF3 genes exist only in the avian herpesvirus genome, they are collectively termed the unique genes of avian herpesviruses. However, few investigations have focused on the functions of these unique genes, particularly LORF5.

In this study, we constructed the LORF5 gene-deletion virus (CHv-BAC-ΔLORF5) and its revertant virus (CHv-BAC-RΔLORF5) using a scarless Red recombination system. Moreover, our data revealed that LORF5 gene is non-essential for DPV proliferation *in vitro*, and dispensable for virus invasion, replication, assembly, and release formation but contributes to the cell-to-cell transmission of DPV.

## Results

### Construction of LORF5 Gene-Deletion Virus and Its Complementing Virus

**FIGURE 1:**
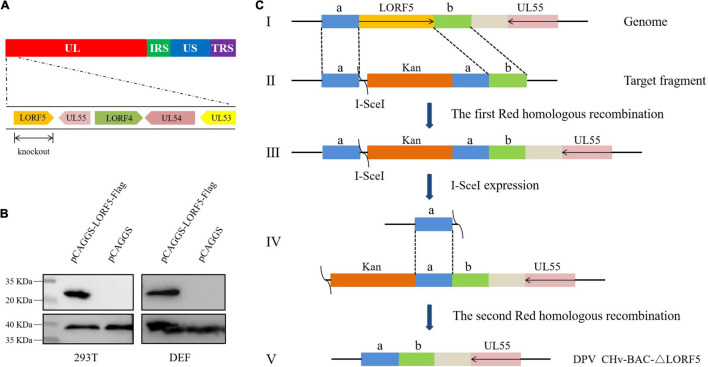
Schematic diagram of CHv-BAC-ΔLORF5 construction. **(A)** DPV genome structure. **(B)** Detection of protein expression of LORF5 gene in eukaryotic plasmid-transfected cells by western blotting. 293T and DEF cells were transfected and harvested at 24 hpi (293T) and 36 hpi (DEF cells). Proteins were detected using a mouse anti-FLAG MAb. **(C)** The principle of knocking out the DPV LORF5 gene. In the first step of homologous recombination, the LORF5 gene was replaced by the Kan resistance gene through 40-bp homology arms (sequences a and b). In the second step, L-arabinose induced recombinase to recognize the I-*Sec*I cleavage site and delete the Kan fragment. Then, the LORF5 gene ORF was deleted in the DPV genome without any reservation.

**FIGURE 2:**
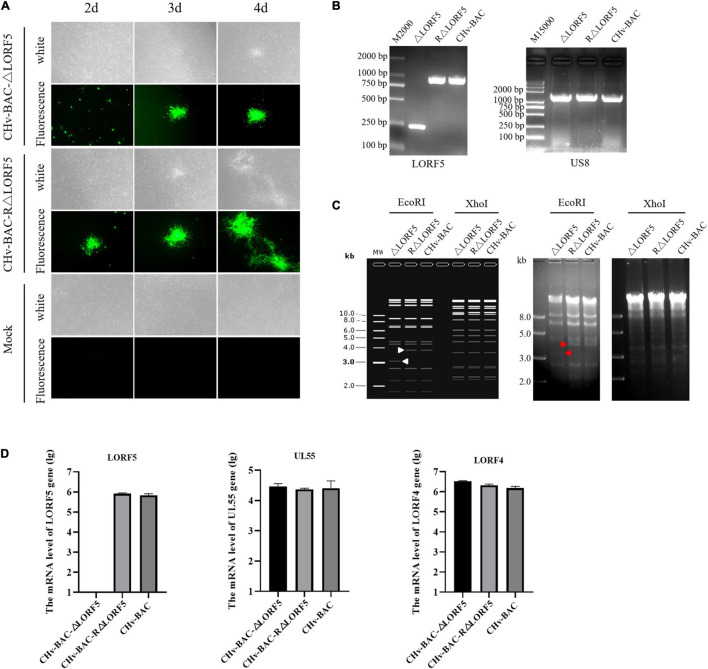
Construction and identification of recombinant viruses. **(A)** Rescue of the LORF5-deleted mutant and its revertant virus. Plasmids from a positive colony were transfected into DEF cells by Lipofectamine 3000, and with continuous observation, the recombinant virus fluorescent marker protein EGFP was expressed in DEFs. **(B)** PCR identification of LORF5 gene deletion (245 bp) or restoration (968 bp) using primers ΔLORF5-F and ΔLORF5-R compared with the parental virus DPV CHv-BAC and the US8 gene (1,473 bp) as a DPV gene control. **(C)** RFLP analysis. The ΔLORF5, RΔLORF5, and BAC plasmids extracted by the Qiagen Plasmid Midi Kit were cut with restriction enzymes *Eco*RI or *Xho*I and then imaged by 1% gel electrophoresis; the left is the simulated imaging after restriction digestion. The arrows in the figure show the difference between the deletion strain with WT and the reverting strain after digestion. The corresponding LORF5 gene-deleted strain has a band around 4 kb after *Eco*RI digestion, and a band around 3 kb has been added. **(D)** Reverse-transcription q-PCR was performed to verify the mRNA expression of the gene LORF5 and surrounding genes UL55 and LORF4 of the viruses.

To determine the role of LORF5 gene in virus replication, a DPV ΔLORF5 mutant was generated on the basis of DPV CHv-BAC, following the method of two-step Red recombination for constructing point mutations reported by Tischer et al. (2010; Figure 1). After transfection of the constructed infectious clone plasmids into DEFs, green fluorescent spots with matching cytopathic lesions were observed in DEF cells on the third day, and they could be passaged stably in new DEF cells, indicating that the LORF5 deletion mutant (CHv-BAC-ΔLORF5) was successfully rescued. Similarly, the revertant virus (CHv-BAC-RΔLORF5) was successfully rescued and harvested (Figure 2A). To confirm the abrogation of LORF5 gene, viral DNA was extracted for PCR analysis (Figure 2B) and positive bacterial artificial chromosome (BAC) clones were confirmed by restriction fragment length polymorphism (RFLP) analysis (Figure 2C) and Sanger sequencing. The plasmid digestion map showed that the bands (right) produced after ΔLORF5, RΔLORF5, and CHv-BAC were digested with *Xho*I or *Eco*RI were consistent with the expected bands (left) of the digestion map. Specifically, the corresponding LORF5 gene-deleted strain has a band around 4 kb after *Eco*RI digestion, and a band around 3 kb has been added (as shown by the arrows). There is no difference in the bands of ΔLORF5, RΔLORF5, and CHv-BAC after *Xho*I digestion. Then, we analyzed ΔLORF5, RΔLORF5, and CHv-infected DEF by quantitative real-time PCR (q-PCR) (Figure 2D), production of the LORF5 mRNA was completely abrogated in ΔLORF5-infected cells, and the transcription of neighboring genes UL55 and LORF4 was not affected. We have identified the extracellular virion protein content by mass spectrometry previously. The results showed that the LORF5 protein was present in mature extracellular virions. Five unique DPV LORF5 peptides were detected, while one unique peptide matched DPV gE (*P* < 0.05). The relative abundance of LORF5 was low based on the exponentially modified protein abundance index (emPAI) (Table 1).

**TABLE 1:** Viral content of DPV extracellular virions (partial).

**Protein**	**Information**	**Score**	**Mass**	**Matches**	**Sequences**	**emPAI**	**NCBI accession**
US8	Glycoprotein E	25	54,873	1 (1)	1 (1)	0.06	ADU04078
LORF5	Virion protein	61	106,375	11 (5)	10 (5)	0.16	AJG04870.1

### Duck Plague Virus LORF5 Slightly Inhibits Virus Replication *in vitro*

**FIGURE 3:**
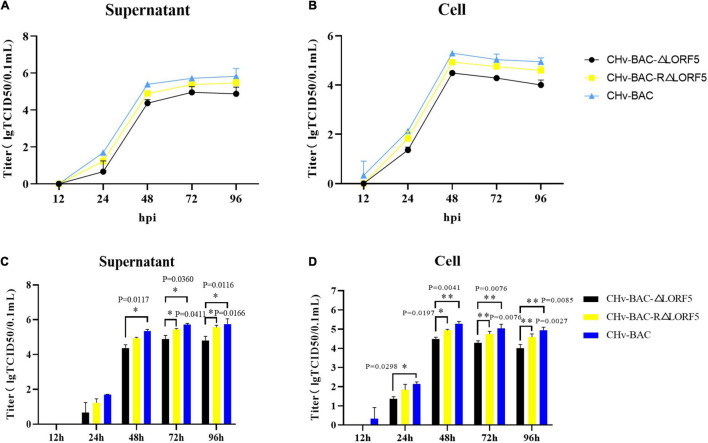
Determination of viral titers in growth kinetics of CHv-BAC-ΔLORF5, CHv-BAC-RΔLORF5, and CHv-BAC. DEF cells in 12-well plates were infected with CHv-BAC, CHv-BAC-RΔLORF5, and CHv-BAC-ΔLORF5 (MOI = 0.01). Samples were collected at the indicated time points, and viral titers were determined. The data were presented as the mean ± standard deviation (SD, *P* > 0.05) of three independent experiments. **(A)** Supernatant viral titers. **(B)** Cell viral titers. **(C)** Statistical analysis of the difference of virus titer in the supernatant at each time point. **(D)** Statistical analysis of the difference of virus titer in the cell at each time point. Asterisks indicate significant differences compared to WT virus (^∗∗^*P* < 0.01; ^∗^*P* < 0.05).

To study whether LORF5 affects the production of mature DPV particles, we investigated if LORF5 plays a role in DPV replication through multistep growth kinetics. Growth kinetics revealed that abrogation of LORF5 slightly inhibits virus replication *in vitro* compared to the wild-type (WT, CHv-BAC) and revertant viruses (Figures 3A,B). Infectious particles were not detected at 12 hpi, which may be related to the latent infection characteristics of DPV. A rapid increase in virus particles was observed during the period of 24–48 hpi, indicating that the replication efficiency of the virus was the highest during this period, entering a slow stage at 48–96 hpi, but the virus titer reached a peak and entered a plateau. Next, we analyzed the difference of the data using GraphPad Prism version 8 (San Diego, CA, United States) and found that regardless of the supernatant (Figure 3C) or intracellular (Figure 3D) source, the viral titers of the LORF5-deleted mutant were lower than those of the parental virus from 24 hpi, especially during the 48–96-h period; the virus titer in the cell decreases more obviously with the difference of two asterisks (^∗∗^*P* < 0.01), and the growth efficiency was restored to the level of the parental virus by reintroducing LORF5 in the revertant virus. We concluded from the results that abrogation of the LORF5 gene slightly but significantly impaired viral proliferation *in vitro* compared to the WT.

### LORF5 Has No Connection With Virus Adsorption and Invasion of Cells

To explore how the LORF5 gene affects the proliferation of the virus *in vitro*, we tested the ability of the LORF5-deleted virus to adsorb cells, invade cells, replicate in the nucleus, and assemble and release virus particles and the infectivity of the virus to spread from cell to cell.

**FIGURE 4:**
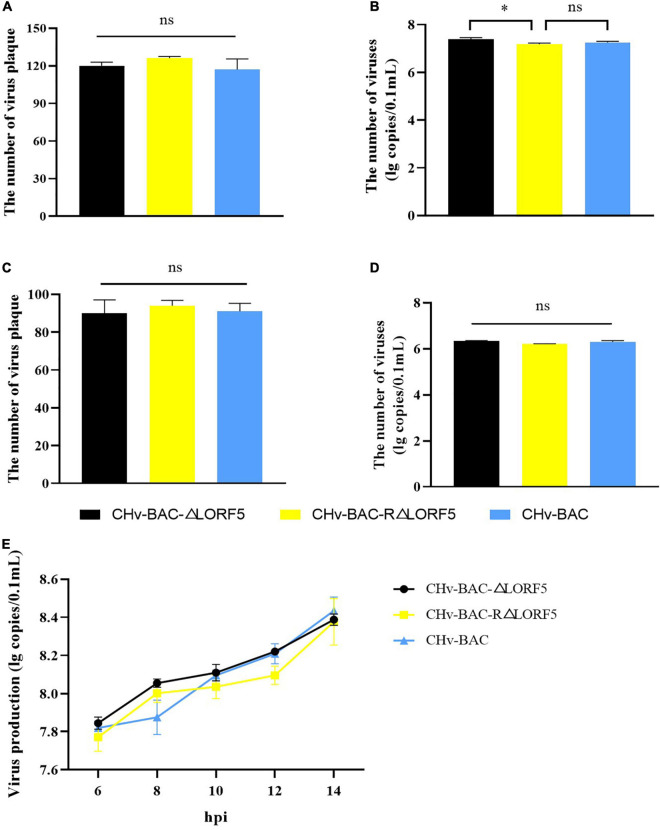
The influence of LORF5 on virus adsorption, invasion, and replication. ΔLORF5, RΔLORF5, and CHv-BAC (0.001 MOI) were inoculated into DEF cells. On the one hand, the number of virus plaques was calculated to study the ability to adsorb on the cell surface **(A)** or invade cells **(C)** after 24 hpi; on the other hand, cell samples were collected 0–2 h after incubating viruses, and the copies were separately detected simultaneously **(B,D)**. **(E)** Cell samples infected with ΔLORF5, RΔLORF5, and CHv-BAC (1 MOI) were collected, RNAs were extracted and reverse-transcribed into cDNA as a template to detect the virus copy number by q-PCR (*t*-test, ^∗^*P* < 0.05).

First, we investigated whether LORF5 acts on virus-adsorbed cells, and the results showed that the number of plaques of the three viruses was almost the same, and there was no significant difference (Figure 4A). On the other hand, the virus copies tested also obtained the same result (Figure 4B). It was concluded that the presence or absence of LORF5 has no effect on the adsorption of virus particles on the cell surface. Similarly, we tested the efficiency of the deletion strain invading the cell, and the results showed that there was no significant difference in the number of viral plaques in ΔLORF5, RΔLORF5, and CHv-BAC (Figure 4C), and the copies of the three viruses were roughly the same (Figure 4D), which was consistent with the results of Figures 4A,B, indicating that LORF5 is not related to DPV infection in cells.

### LORF5 Has No Connection With the Replication of the Viral Genome

Next, to explore the effect of LORF5 on DPV genome replication, ΔLORF5, RΔLORF5, and CHV-BAC [multiplicity of infection (MOI) = 1] were separately inoculated into DEF cells, and cell samples were collected to measure virus copies by q-PCR. The results demonstrated that the viral genome content increased over time; ΔLORF5, RΔLORF5, and CHv-BAC exhibited similar proliferation patterns during this process; and the genomic copies of the three viruses at each time node were almost equal (Figure 4E), explaining why LORF5 does not function in the replication stage of the viral genome.

### LORF5 Does Not Promote the Assembly and Release of Virus Particles

**FIGURE 5:**
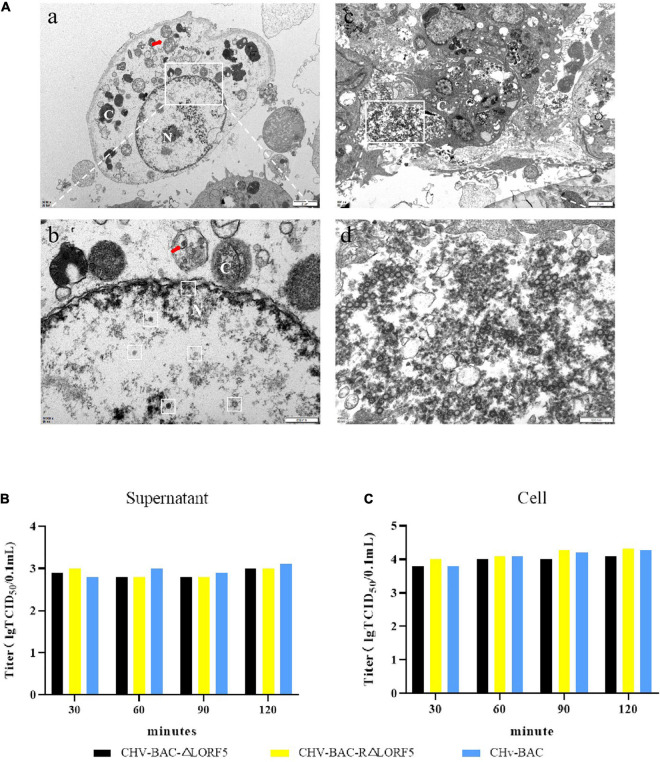
The role of LORF5 in the assembly and release of virus particles. **(A)** Electron microscopy analysis of DEF cells infected with CHv-BAC-ΔLORF5. DEF cells were infected with 5 MOI of virus and examined by electron microscopy analysis (N, nucleus; C, cytoplasm). The white box in the middle indicates virus particles. **(B)** Eighteen hours after the cells were infected with the viruses, the medium was replaced with 2% maintenance solution; the supernatant was collected at 30, 60, 90, and 120 min after changing; and the infectious mature virus particles in the supernatant were detected. **(C)** At the same time, the cells were used as control. The means and standard deviations were measured with GraphPad Prism 8. Standard deviations are shown by error bars.

In that way, will knocking out LORF5 affect the maturation of virus particles? Therefore, we observed the replication of ΔLORF5 in DEFs through transmission electron microscopy. In Figure 5A, a quantitative analysis of virions at different morphogenetic stages was performed using infected cells that were randomly observed under low-magnification electron microscopy. The data presented in Table 2 show the percentages of virions in different morphogenetic stages. The arrow shows the vesicles that reassembled the viruses, and such vesicles were abundant in the cytoplasm. Nucleocapsids and empty capsids were observed in the nucleus, of which nucleocapsids accounted for 66.7%, and there was no obvious perinuclear aggregation (Figures 5Ab). Moreover, we observed that the cells lysed after infection exhibited many soluble vacuoles, and the rupture of the cell membrane released a large amount of virus in the intercellular space (Figures 5Ac,d). Simultaneously, we infected DEF cells with 1-MOI virus, detected the titer of infectious virus particles released into the supernatant, and found that there was no significant difference in the mature virus particles in the supernatant (Figure 5B), while the viral titers in the cells of the deletion strain, the revertant strain, and the parent strain were at the same level (Figure 5C). The data show that LORF5 has no effect on the assembly and release of virus particles. Based on the above experiments, we can conclude that the presence or absence of LORF5 does not affect the adsorption and invasion of the virus or the replication, assembly, and release of the viral genome *in vitro*.

**TABLE 2:** Virions observed in ΔLORF5-infected DEF cells by electron microscopy.

**% of virions in different morphogenetic stages (particles in a stage)**					
**Nucleus**		**Total counted (virion/cells)**	**Extracellular**		**Total counted (virion/cells)**
**Intranuclear**	**Perinuclear area**		**Mature virus**	**Immature virus**	

66.7% (28)	33.3% (14)	42/4	67.6% (75)	32.4% (36)	111/2

### LORF5 Played an Important Role in the Spread of Viruses From Cell to Cell

Then, we investigated if LORF5 plays a role in DPV replication through plaque size assays. As introduced in Materials and Methods, a 1.5% methylcellulose semisolid cell culture medium was used to ensure that adjacent cells were infected only by viral cell-to-cell spread; then we tested the transmission of deletion virus, WT, and reverted virus through plaque experiments.

**FIGURE 6:**
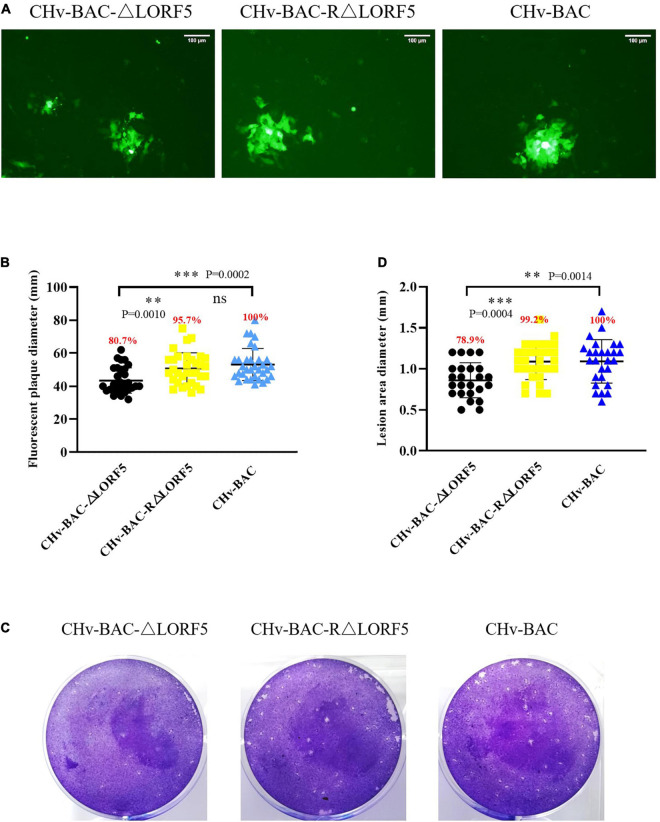
Plaque size assays of indicated recombinant viruses. DEF cells in six-well plates were infected with 0.001 MOI of ΔLORF5, RΔLORF5, or CHv-BAC. After incubation at 37°C for 2 h, the infected cells were covered with 1.5% methylcellulose and cultured in a 37°C, 5% CO_2_ incubator. **(A)** Green fluorescent plaques produced by ΔLORF5, RΔLORF5, and CHv-BAC. The cells were observed under a fluorescence microscope (Nikon TI-SR, Japan). **(B)** Statistical analysis of the data in panel **(A)** and shown as a scatter plot with minimum and maximum values (plaque diameters: mm). **(C)** Images of viral plaques after 0.5% crystal violet staining. **(D)** Statistical analysis of the data in panel **(C)** and shown as a scatter plot with minimum and maximum values. All data have been carried out in three independent experiments. The plaque size of the deletion virus and the reverted virus was compared with that of the parental virus (WT, CHv-BAC) set to 100%. Asterisks indicate significant differences compared to WT virus (^∗∗∗^*P* < 0.001; ^∗∗^*P* < 0.01; *n* > 50). The means and standard deviations were measured with GraphPad Prism 8. Standard deviations are shown by error bars.

Here, we used two methods for plaque statistics. In the first method, we detected the area of fluorescent plaques produced by ΔLORF5, RΔLORF5, and CHv-BAC in infected cells at 36 h (Figure 6A). The fluorescent area formed by the ΔLORF5 mutant virus was smaller than that of RΔLORF5 and CHv-BAC. On average, the diameter of the fluorescent plaque formed by the ΔLORF5 strain was 43.6 mm, while that of RΔLORF5 was 51.7 mm and that of CHv-BAC was 54.0 mm. And Figure 6B was the statistical analysis of Figure 6A; abrogation of LORF5 slightly impaired viral spread by 19.3% compared with WT (100%). In the second method, we counted the cytopathic plaques by crystal violet staining when the cells were infected for 5 days (Figure 6C). The average size of plaques produced by ΔLORF5 was 0.860 mm, while RΔLORF5 was 1.087 mm and CHv-BAC was 1.090 mm. And Figure 6D was the statistical analysis of Figure 6C; the size of the plaques formed by ΔLORF5 was reduced by 21.1% compared with WT, and there was no difference between WT and the reverting strain (data were considered significantly different if the *P*-value was ≤0.05). Then we come to the conclusion that the loss of the LORF5 gene truly reduces the transmission efficiency of DPV between DEF cells. In other words, we found that LORF5 has a function in cell-to-cell transmission, which was the first report.

## Discussion

Unlike other herpesviruses, MDV has some genes which are unique to the *Mardivirus* genus, such as Meq and pp38, which play an important role in pathogenicity and oncogenicity. However, the genes of the LORF series are also unique based on sequence alignment. Until now, it remains unknown whether these putative avian herpesvirus-specific genes are translated into proteins and what their role is in the viral life cycle. Constructing a DPV CHv-BAC-ΔLORF5 recombinant virus by applying DPV CHv-BAC, with its biological properties being compared with those of the replenishment plant and parental strain, we concluded that the ΔLORF5 recombinant virus exhibited a similar growth pattern on the cells as the complement strain and parent strain, although its proliferation was slightly lower than that of RΔLORF5 and CHv-BAC (Figure 3). Tests related to the life cycle of the virus found that the LORF5 gene has no significant effect on the adsorption, invasion, replication, and release of the virus, while the plaque assay showed that the fluorescent plaque area of the ΔLORF5 recombinant virus was significantly reduced and the cell-to-cell transmission efficiency was significantly reduced (Figure 6B). These results revealed for the first time that the LORF5 gene is not necessary for the replication of DPV CHv, but it is important for the spread of DPV CHv in host cells.

In this article, CHv-BAC-ΔLORF5 was generated on the basis of CHv-BAC, which was constructed by Ying Wu et al. (2017). BACs were developed in the 1990s and can be used to construct genome-wide operating systems for large-genome viruses such as herpesviruses. With the rapid development of BAC technology, BAC infectious clones have been successfully constructed for a variety of herpesviruses, such as murine cytomegalovirus (MCMV) (Messerle and Koszinowski, 1997), Marek’s disease virus serotype 1 (MDV-1) (Schumacher et al., 2000), human cytomegalovirus (HCMV) (Marchini et al., 2001), herpes simplex virus type 1 (HSV-1) (Kuroda et al., 2006), DPV CHv (Wu et al., 2017), and equine herpes virus serotype 3 (EHV-3) (Akhmedzhanov et al., 2017). BAC infectious clones have been widely employed in the construction of viral gene deletions. For example, DPV CHv-BAC has been successfully applied to the construction of several recombinant viruses (Wu et al., 2017; You et al., 2018) and laid the foundation for this experiment.

It is worth mentioning that apart from basic information such as gene sequences and the number of encoded amino acids, there are few reports on LORF5 functions. To our knowledge, previous research on LORF5 function has been performed only in the MDV-1 Md5 strain, in which it was found that the LORF5 gene is non-essential for MDV Md5 replication in cells (Lee et al., 2007). In addition, the MDV-1 Md5 ΔLORF5 recombinant virus exerted no tumorigenicity in chickens, and the mortality rate was significantly lower than that of the parent strain, suggesting that the LORF5 gene might be a virulence gene of MDV-1 (Lee et al., 2007). However, the LORF5 gene is highly variable, and the similarity among the LORF5 genes in different viruses is very low; thus, further research is required to determine whether the LORF5 gene of other viruses is related to virulence.

In this study, we investigated this novel LORF5 gene in the context of virus replication. Using the △LORF5 mutant virus, we could demonstrate that the deletion of LORF5 slightly, but significantly, affects viral proliferation and that LORF5 gene is non-essential for the replication of DPV CHv, which is consistent with findings for the MDV-1 LORF5 gene. Furthermore, we focused on the LORF5 gene function in DPV CHv cell-to-cell spread for the first time and found that it plays a positive role in this process. Previous studies have shown that most of the proteins involved in herpesvirus cell-to-cell spread are envelope glycoproteins, such as gE, gI, gH, gB, gL, gQ, gJ, and gM (Ziegler et al., 2005; Farnsworth and Johnson, 2006; Tanaka et al., 2013; Howard et al., 2014; You et al., 2018). Among them, the gE and gI glycoproteins, which usually act as functional units, are critical for this process in α-herpesviruses, and in-depth mechanistic studies have been reported on this topic (Damiani et al., 2000; Johnson et al., 2001; Collins and Johnson, 2003; Devlin et al., 2006). Although DPV and HSV-1 belong to the same family of herpesviruses, the study of DPV is far behind that of HSV-1. For example, in DPV, only the gJ gene has been confirmed to be associated with cell-to-cell spread (You et al., 2017). The results of our study suggest that the LORF5 gene plays a positive role in the cell-to-cell spread of DPV CHv in DEF cells. However, since little research has been reported on the DPV LORF5 gene, its protein properties are unclear, and the mechanism by which it functions is not known. It remains unclear whether the LORF5 gene directly or indirectly affects the cell-to-cell spread of DPV CHv and whether it can also promote the transmission of DPV in nerve cells; moreover, the proteins that interact in this process are unknown. More in-depth studies are required to explore these issues.

In conclusion, we have identified that the LORF5 gene is not essential for virus replication *in vitro*. In addition, we demonstrate that pLORF5 does not affect virus invasion, replication, assembly, and release formation but plays a positive role in cell-to-cell spread. Our results provide insights for in-depth studies of LORF5 gene functions.

## Materials and Methods

### Ethics Statement

All duck embryo experiments were approved by the Committee of Experimental Operational Guidelines and Animal Welfare of Sichuan Agricultural University, with approval number S20167031-1707. Experiments were conducted in accordance with approved guidelines.

### Cells, Viruses, and Primers

DEF cells were prepared from 9- to 12-day-old unfertilized duck embryos containing 10% serum MEM (serum, MEM from Gibco) as the growth medium and 2% serum as a maintenance medium. The DPV CHv strain was separated and preserved in the laboratory (accession no. JQ647509). All primers used in this paper were designed by Primer 5 software. The primer sequences and products are shown in Table 3.

**TABLE 3:** Primers used in this paper.

**Name**	**Sequence (5′–3′)**	**Product**
ΔLORF5-Kan-F	cgtactaactgaagttgaatctgatcta gtaaccggggcctagggataacagggta atcgatttatgtgaaagacgtcaaaagttaaa accggtatattaaatgggccccggtt	Target ΔLORF5 fragment
ΔLORF5-Kan-R	actagatcagattcaacttcagttagtacggcca gtgttacaaccaat	
ΔLORF5-F	gccaacggtagggactg	ΔLORF5/RΔLORF5 identification
ΔLORF5-R	gacacggtaaacaatgaagg	
RΔLORF5-F	aacagatgtccgtatgtaatcgtactaactg aagttgaatctgatctagtaaccg gggccatggcatcttcaaaagcgtt	Target RΔLORF5 fragment
RΔLORF5-R	aaagttaaaaccggtatattaaatgctaata gtcatctctggtat	
RΔLORF5-Kan-F	catttaatataccggtttt aacttttgacgtctttcacattacttaaatat agggataacagggtaatcgat	
RΔLORF5-Kan-R	cgagactgattcgtttaatcatatttatttt atttaagtaatgtgaaagacgtcaaaagtt aaaaccggtatattaaatgt gttacaaccaattaacc	
US8-F	tctcaagacgctctggaatc	US8 (gE)
US8-R	gacgcagagaagtactcgct	
LORF5-F	aacgactggtgggagtaacg	LORF5
LORF5-R	gcagcggaacaaatgaaac	
LORF4-F	cgcaccctatgctatcgtc	LORF4
LORF4-R	cgttgtcggattacccattt	
UL55-F	tattcttctgcgggctca	UL55
UL55-R	catagacgatgctcc	
UL30-F	tttcctcctcctcgctgagtg	UL30
UL30-R	ccagaaacatactgtgagagt	

### Construction of Mutant Strains Using a Two-Step Red Recombination

GS1783-pBAC-DPV was constructed by Ying Wu in our laboratory, in which the DPV genome was cloned into a BAC for the goal of DPV modification in bacteria. The targeted fragments were amplified using primers (Table 3) and then targeted into *Escherichia coli* GS1783-pBAC-DPV competent cells. In the first step of homologous recombination, the LORF5 gene was replaced by the Kan resistance gene (1,354 bp). Then, the I-*Sec*I site was cleaved followed by a second homologous recombination, resulting in the deletion of the Kan fragment (245 bp). To obtain the mutant viruses, the ΔLORF5 or RΔLORF5 plasmid (1 μg) using the QIAGEN Plasmid Midi Kit (cat. no. 12143) from a positive colony was transfected into DEF cells by Lipofectamine^®^ 3000 Invitrogen (L3000001), the MEM medium with 2% calf serum was replaced, and the culture was continued at 37°C after incubating for 6 h. PCR analysis was performed on fourth-generation virus DNA with primers of US8 and LORF5 (Table 3). Positive BAC clones extracted with the Qiagen Plasmid Midi Kit were confirmed by RFLP analysis, with the system (25 μL) including 1 μg plasmid, 2 μL restriction enzyme *Eco*RI, 4 μL 10× Q.Cut G.Buffer, and ddH_2_O to replenish, analyzed by 1% agarose gel electrophoresis at 50 V electrophoresis for 2–4 hpi after cutting at 37°C for 2 hpi.

### Multistep Viral Growth Kinetics

DEF cells in 12-well plates were infected with 0.01 MOI of CHv-BAC-ΔLORF5, CHv-BAC-RΔLORF5, or CHv-BAC; the plates were shaken every 15 min during the 37°C incubation for 2 h; and then the MEM medium with 2% calf serum was changed. Samples of the infected cells and their supernatants were collected separately at 12, 24, 48, 72, and 96 hpi, and the volume of each sample was increased to 1,000 μL with MEM, and then the samples were frozen and thawed three times. Intracellular viral titers and supernatant viral titers were detected by determining the 50% tissue culture infectious dose (TCID_50_) to assess the virus. Statistical analyses were performed using GraphPad Prism version 8 (San Diego, CA, United States), and data were considered significantly different if the *P*-value was ≤0.05. Growth kinetics data were repeated three independent times. Asterisks indicate significant differences compared to WT virus (^∗∗∗^*P* < 0.001; ^∗∗^*P* < 0.01, ^∗^*P* < 0.1).

### Virus Adsorption and Invasion Experiments

Culture DEF cells in a monolayer were placed in a six-well plate. After the cells were full for 12–24 h, they were precooled at 4°C for 1 h, washed with 4°C precooled PBS buffer to remove dead cell debris, then infected with 0.001 MOI of virus, incubated at 4°C for 2 h (agitated every 5 min), and then washed five times with precooled PBS. The viral genome was extracted from the cell samples (TaKaRa MiniBEST Viral RNA/DNA Extraction Kit Ver. 5.0), and the copies were detected by q-PCR. Alternatively, the medium was replaced with 1.5% solid medium after washing, and the number of plaques was counted after 36 h of incubation at 37°C. To detect invasion, cells were washed after incubation at 4°C, incubated at 37°C for 1–2 h, and then subjected to the same experiments. The number of plaques was calculated and analyzed by GraphPad Prism 8.0.2 software.

### RNA Extraction and Reverse-Transcription Quantitative Real-Time PCR Analysis

Total RNA was isolated from virus-infected cells using the RNeasy Plus Mini Kit (Qiagen, Hilden, Germany) according to the manufacturer’s instructions. DNase I (Promega, Fitchburg, WI, United States) was used to remove any genomic DNA, and then cDNA was generated using the high-capacity cDNA reverse-transcription kit (Applied Biosystems/Thermo Fisher Scientific, Waltham, MA, United States). Premix Ex Taq^TM^ (Probe q-PCR) (Takara, Dalian, China) was used to determine viral cDNA copies. The primers and probe used to detect the BAC-CHv UL30 gene by q-PCR were designed previously in our laboratory. q-PCR amplifications were performed under the following conditions: 95°C for 30 s, followed by 40 cycles at 95°C for 5 s and 60°C for 30 s. Then, the q-PCR products were quantified by comparison with the established standard curve of the laboratory. For mRNA transcription level, the LORF5, UL55, and LORF4 genes were detected using the previously described primers. The conditions were set as follows: initial denaturation at 95°C for 1 min, followed by 45 cycles of denaturation at 95°C for 5 s, annealing at 59°C for 20 s, and extension at 72°C for 25 s. All reactions were performed in triplicate and in at least three independent experiments. The cycle number at threshold (Ct value) was determined to analyze these gene transcriptions, and the results were calculated using the 2^–ΔΔCt^ method.

### Electron Microscopy Analysis of Recombinant Viruses

DEF cells in 60-mm dishes were infected with CHv-BAC-ΔLORF5 at an MOI of 5, collected in a centrifuge tube by scraping at 14 hpi, and centrifuged at 1,000 rpm for 5 min to collect the cells; and the supernatant was discarded. The cells were added with 0.5% glutaraldehyde fixative solution, resuspended, and fixed for 10 min at 4°C; then centrifuged at 12,000 rpm for 10 min; and then readded with 3% glutaraldehyde fixative solution for fixation. All samples were then sent to Chengdu Lilai Biological Technology Co., Ltd., for analysis under a transmission electron microscope (Hitachi H-7650, Tokyo, Japan).

### The Plaque Morphology of Recombinant Viruses

DEF cells in six-well plates were infected with 0.001 MOI of CHv-BAC-ΔLORF5, CHv-BAC-RΔLORF5, or CHv-BAC. After incubation for 2 h at 37°C, 1.5% methylcellulose (Solarbio, Beijing, China) was added to cover the cells. Here, we used two methods for plaque statistics. In the first method, the green fluorescent plaques produced by ΔLORF5, RΔLORF5, or CHv-BAC-infected cells at 36 h were calculated, and the cells were observed under a fluorescence microscope (Nikon TI-SR, Japan). Fifty randomly selected green fluorescent plaques were photographed per experiment. The second method counted the cytopathic plaques by crystal violet staining when the cells were infected for 5 days (cultured in a 37°C, 5% CO_2_ incubator). In particular, the medium was discarded, and cells were fixed with 500 μL of precooled 4% paraformaldehyde at room temperature for 20 min, washed twice with sterile PBS, and added with 500 μL 0.5% crystal violet for staining for 30 min; they were then rinsed with tap water to remove the staining solution and to observe and count the plaques. All the average plaque size was measured using Image-Pro Plus software (Bio-Rad, CA, United States). The plaque size of the deletion virus and the reverted virus was calculated and compared with the plaque size of the parental virus set at 100%. Data were considered significantly different if the *P*-value was ≤0.05 (^∗∗^*P* < 0.01, ^∗∗∗^*P* < 0.001). All reactions were performed in triplicate and in at least three independent experiments.

## Data Availability Statement

The original contributions presented in the study are included in the article/Supplementary Material, further inquiries can be directed to the corresponding author.

## Author Contributions

BS and YunjL carried out the experiments. AC and MW conceived and supervised the study. MW, YW, QY, RJ, BT, XO, SM, DS, SZ, DZ, and SC provided ideas contributing to the conception of this article. ML, X-XZ, JH, and QG helped to draw the pictures. MW modified the article. All authors reviewed the manuscript.

## Conflict of Interest

The authors declare that the research was conducted in the absence of any commercial or financial relationships that could be construed as a potential conflict of interest.

## Publisher’s Note

All claims expressed in this article are solely those of the authors and do not necessarily represent those of their affiliated organizations, or those of the publisher, the editors and the reviewers. Any product that may be evaluated in this article, or claim that may be made by its manufacturer, is not guaranteed or endorsed by the publisher.
