# Supplementary material for: The LORF5 Gene Is Non-essential for Replication but Important for Duck Plague Virus Cell-to-Cell Spread Efficiently in Host Cells
Source: Front Microbiol. 2021 Dec 2;12:744408. doi: 10.3389/fmicb.2021.744408 (PMC8674210; doi:10.3389/fmicb.2021.744408)

Original image data used in the article

Figure 2B

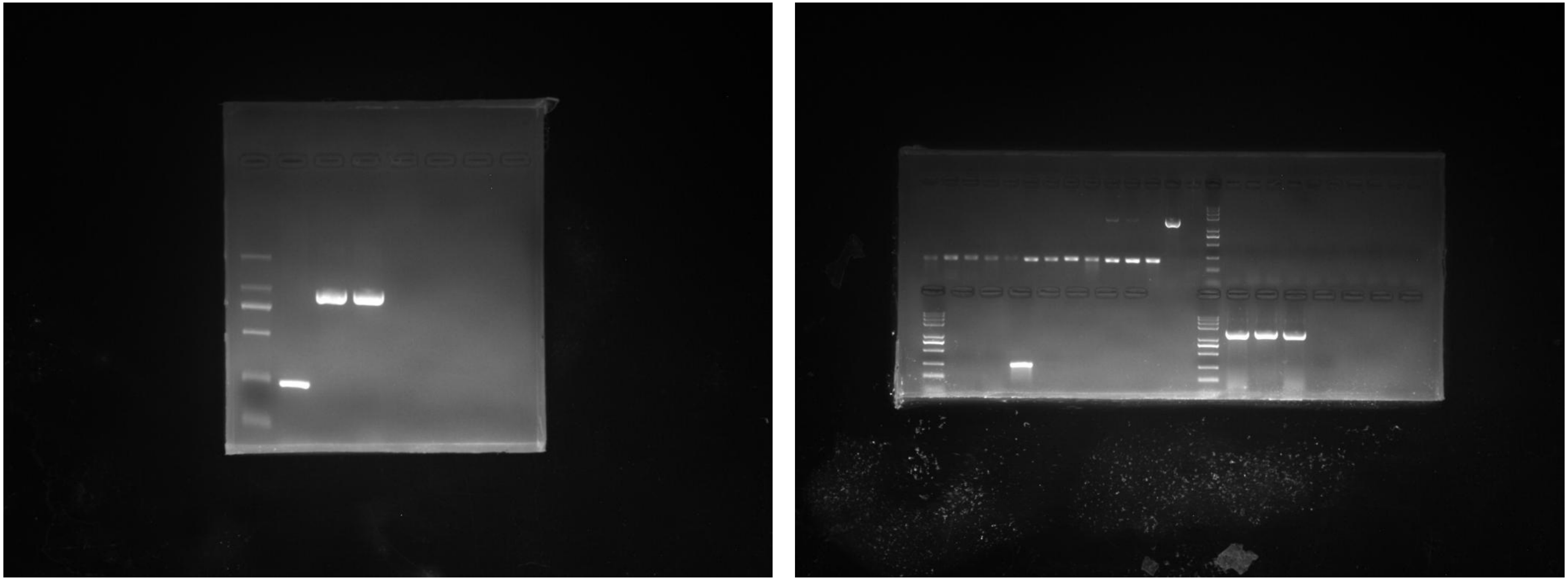

Figure 2C

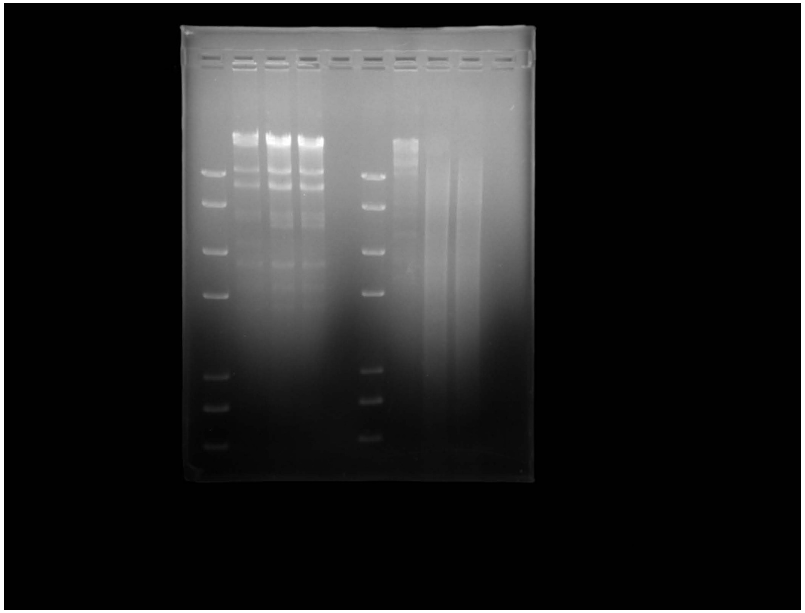

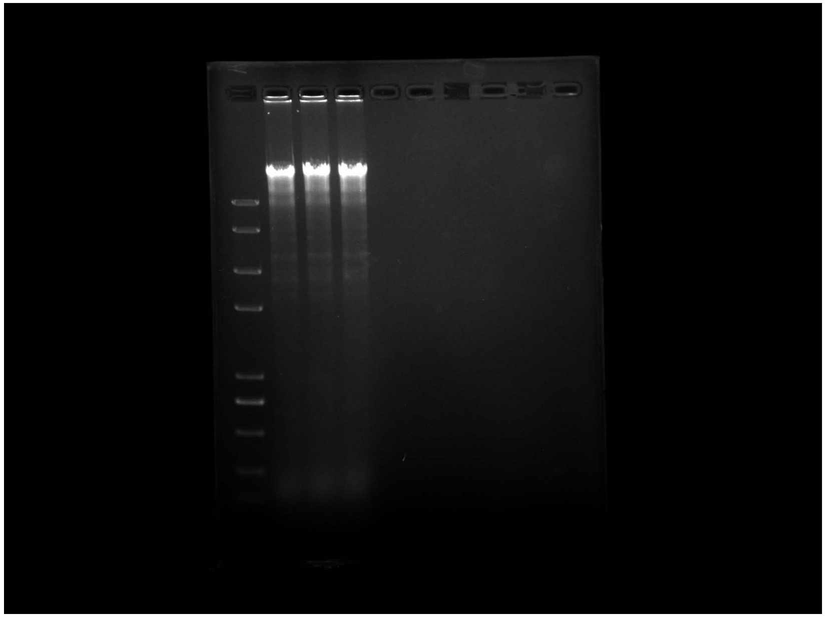

Figure 5A

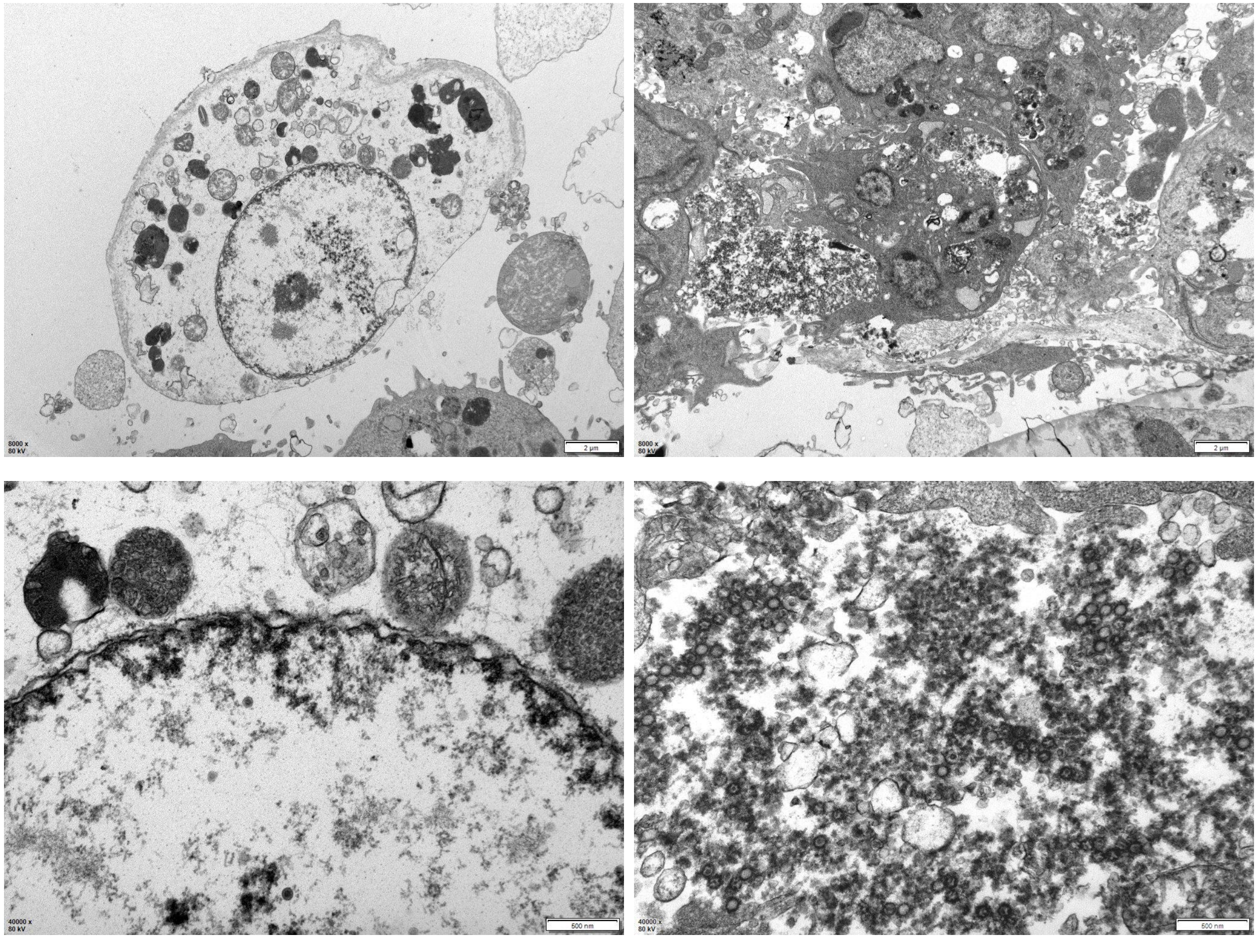
8000 x
80 kV
2 µm
8000 x
80 kV
2 µm
40000 x
80 kV
500 nm
40000 x
80 kV
500 nm

Figure 6 (CHv-BAC-△LORF5)

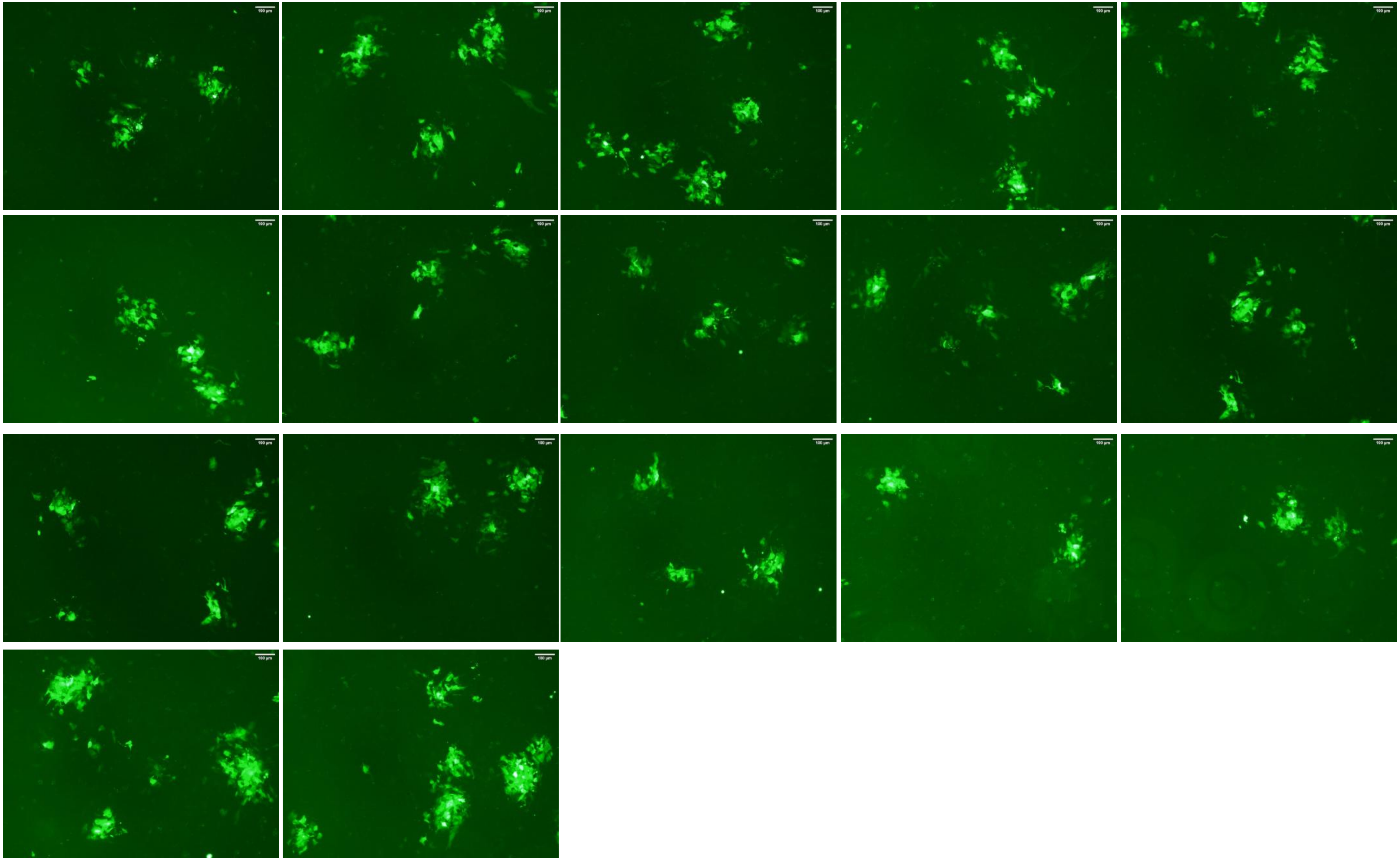

Figure 6 (CHv-BAC-R△LORF5)

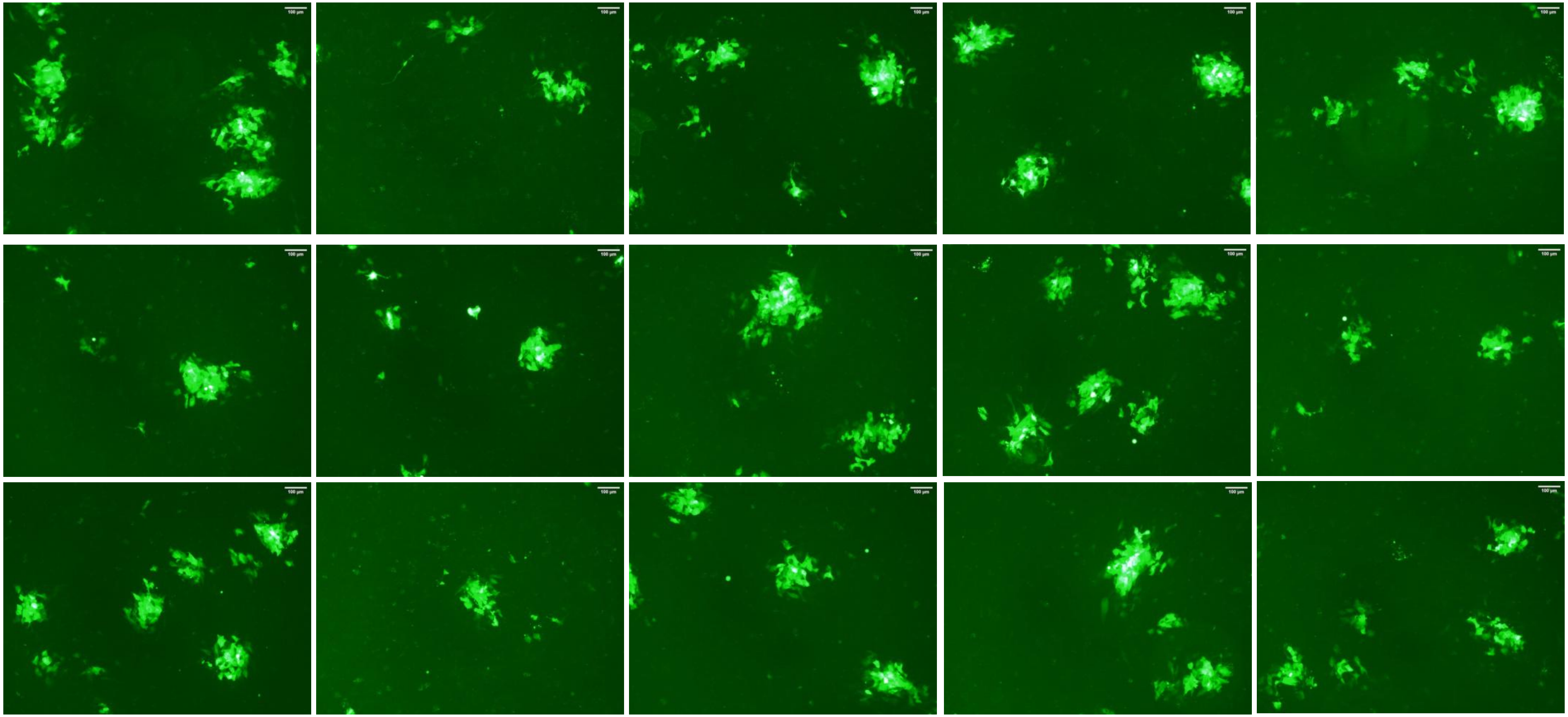

Figure 6 (CHv-BAC)

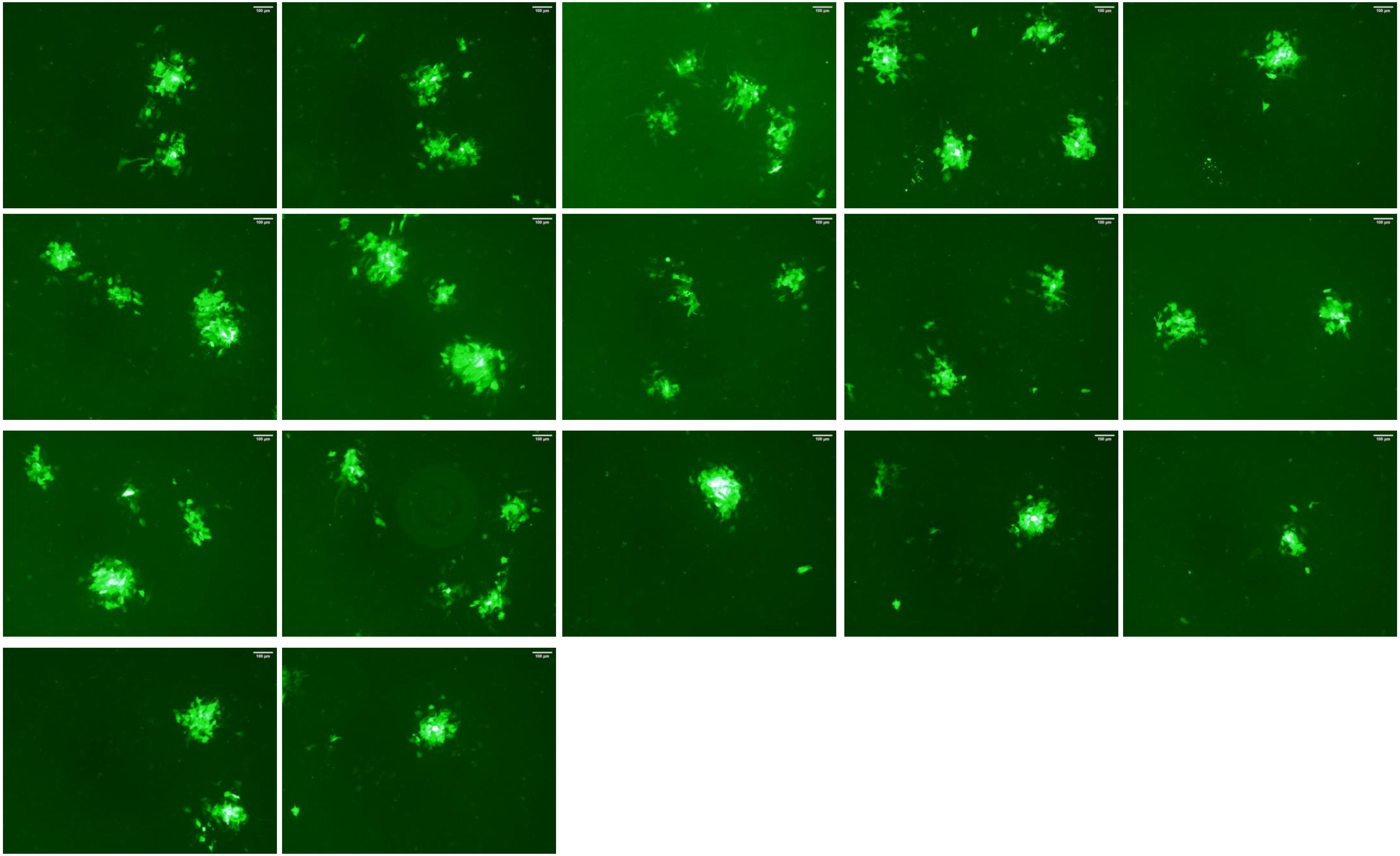

Supplement: Supplementary file 3 [file Data_Sheet_1.pdf]
